# Negative-Pressure Pulmonary Edema: A Perioperative Emergency

**DOI:** 10.7759/cureus.91868

**Published:** 2025-09-08

**Authors:** Nissar Shaikh, Bakri Alali, Umm E Amara, Umm E Nashrah, Firdous Ummunnisa

**Affiliations:** 1 Surgical Intensive Care, Hamad Medical Corporation, Doha, QAT; 2 Clinical Academic Sciences, Qatar University College of Medicine, Doha, QAT; 3 Critical Care, Deccan College of Medical Sciences, Hyderabad, IND

**Keywords:** diuretics, glottis, invasive ventilation, laryngospasm, negative-pressure pulmonary edema, noninvasive ventilation, peep, upper airway, β-agonist

## Abstract

Negative-pressure pulmonary edema (NPPE) is a potentially fatal perioperative condition that requires increased awareness. This narrative review highlights the underrecognized aspects of the condition and analyzes the risk factors, diagnosis, management, and prevention of NPPE in the current literature. NPPE most frequently develops in the post-extubation period. It is a non-cardiogenic and non-fluid-overload pulmonary edema that occurs due to the generation of high negative pressure against a closed glottis, hence also termed obstructive pulmonary edema. NPPE is potentially lethal and requires intensive care therapy. Risk factors for developing NPPE include young age, male sex, and upper airway surgery or obstruction. Clinical manifestations include pink frothy sputum and respiratory distress. Chest X-ray and ultrasound reveal diffuse infiltrates and increased B-lines, respectively. NPPE should be differentiated from cardiogenic pulmonary edema, tension pneumothorax, aspiration pneumonia, and fluid overload by history and monitoring. Milder cases of NPPE improve with oxygen therapy and the use of non-invasive ventilation to maintain airway patency. Severe NPPE cases require intubation and invasive ventilation with positive end-expiratory pressure. β-agonist therapy may help by accelerating alveolar fluid clearance. Severe cases of NPPE with persistent hypoxia may require extracorporeal membrane oxygenation support. Preventive measures include suctioning of endotracheal secretions before extubation, limiting the number of laryngoscopy attempts, considering deep or awake extubation, and the use of lidocaine.

## Introduction and background

Negative-pressure pulmonary edema (NPPE) is lung edema caused by the generation of excessive negative pressure against a closed glottis or upper airway; hence, it is also called obstructive pulmonary edema. It most frequently occurs due to post-extubation laryngospasm in adults, and in children, supraglottic or inspiratory croup is the main etiology of NPPE. The large negative pressure in the airway leads to fluid accumulation in the alveoli, causing oxygenation impairment and pulmonary edema. Although management is supportive and patients usually improve within 24 to 48 hours, NPPE remains a potentially fatal clinical entity [1]. Of note, there is no fluid overload contributing to pulmonary edema in NPPE [1].

The relationship between upper airway obstruction and pulmonary edema was first described in 1927 by Moore and Binger, and Warren et al. in 1942 described the association between negative pressure and the development of pulmonary edema [2]. Oswalt et al. in 1977 demonstrated the immediate occurrence of pulmonary edema after acute severe upper airway obstruction [3]. Over the decades, it has become clear that immediately post-extubation, or the sudden relief of chronic airway obstruction, may lead to the occurrence of NPPE, which is a life-threatening clinical entity requiring emergent acute critical care.

## Review

Methodology

A thorough search was performed using the keyword “negative pressure pulmonary edema” on PubMed, Google Scholar, conference proceedings, and published abstracts. All available literature was kept in an electronic folder and reviewed by the authors, and only NPPE publications were included, excluding the literature about pulmonary edema due to other etiologies.

Epidemiology

NPPE is underreported as the milder cases of NPPE remain unnoticed. The condition frequently occurs in healthy patients, and the milder cases are managed in the operating theater [4-6]. NPPE has been reported to occur in 0.05% to 0.1% of all cases of general anesthesia in the adult population. On the other hand, Tami et al. reported a much higher incidence of 1% of NPPE after general anesthesia [4]. Viswanathan et al. recorded 4,000 incidences of laryngospasm after general anesthesia and found NPPE in only 4% of those patients [5], whereas Deepika et al. reported an incidence of 0.1% of NPPE in cases of laryngospasm [6].

In 176 children with severe upper airway obstruction, the incidence rate of NPPE was 9.6% [7]. In an older study, the incidence of NPPE in patients with acute upper airway obstruction was reported to be as high as 12% [4]. In the Australian Incident Monitoring Study, the incidence rate of NPPE in patients with laryngospasm was 3% [8]. The incidence rate of NPPE has also been reported to be as low as 0.1% in laryngospasm cases [6]. Thus, the estimated incidence rate of NPPE is between 0.1% and 12% [9,10]. However, considering the frequent occurrence of upper airway obstruction during the peri-anesthesia period, the actual incidence rate may be much higher than that reported so far, as several cases are misdiagnosed or overlooked [1]. Moreover, given the frequent occurrence of post-general anesthesia or post-extubation laryngospasm, with a higher frequency of oversight, misdiagnosis, or underreporting of NPPE, the overall incidence appears to be more than that reported in the literature.

Classification

NPPE is divided into two types, depending on the etiology of its occurrence. Type 1 NPPE occurs after laryngospasm and airway obstruction with extreme negative pressure generation. Type 2 NPPE occurs after the relief of chronic upper airway obstruction due to various etiologies. Type 1 NPPE is reported after acute post-extubation and upper airway obstruction to be around 9-12%. In adult patients, around 8% of NPPE cases are due to laryngospasm [11]. Type 2 NPPE is reported in up to 44% of patients with chronic upper airway obstruction relief [4]. More recently, recurrent pulmonary edema has been described [12].

Type I NPPE typically occurs after sudden, intense upper airway obstruction, such as post-extubation laryngospasm. The forceful inspiratory effort against the obstruction creates a large negative intrathoracic pressure, drawing fluid into the lungs. It often develops rapidly, within minutes of the obstruction. Type II NPPE develops after the relief of chronic airway obstruction, for instance, after laryngeal tumor resection or adenoidectomy, huge tonsils and adenoid gland, upper airway tumors, and nasopharyngeal and mediastinal tumors. The relief of the obstruction leads to a loss of positive end-expiratory pressure (PEEP), potentially causing fluid to shift into the lungs. The edema commonly develops after the obstruction is relieved [8].

Etiology and risk factors

Several factors that cause acute or chronic upper airway obstruction can lead to NPPE. Apart from upper airway trauma or laryngospasm, upper airway infections and vocal cord impairments may also cause NPPE. Laryngospasm is the most common etiology for NPPE. The risk factors for laryngospasm are male gender; obesity; obstructive sleep apnea; nasal, nasopharyngeal, and laryngeal surgeries; and difficult intubations [13,14]. In adult patients, post-extubation, free secretions entering the larynx cause laryngospasm, and at the same time, the diaphragm strongly contracts, leading to the development of NPPE. In upper airway infection, particularly croup in the pediatric age group that causes glottic and supraglottic obstructions, when the airway obstruction is suddenly cleared by intubation, NPPE may develop [15] (Table 1).

**Table 1:** Etiology and risk factors.

Type 1	Type 2
Laryngospasm (after general anesthesia)	Post removal of upper airway tumors
Strangulation	Post large tonsil or adenoid removal
Endotracheal tube (obstruction or biting)	Big redundant uvula removal
Supraglottitis	
Croup	
Near drowning	
Migration of the tamponade balloon in the epiglottis	

Pathophysiology

**Figure 1:**
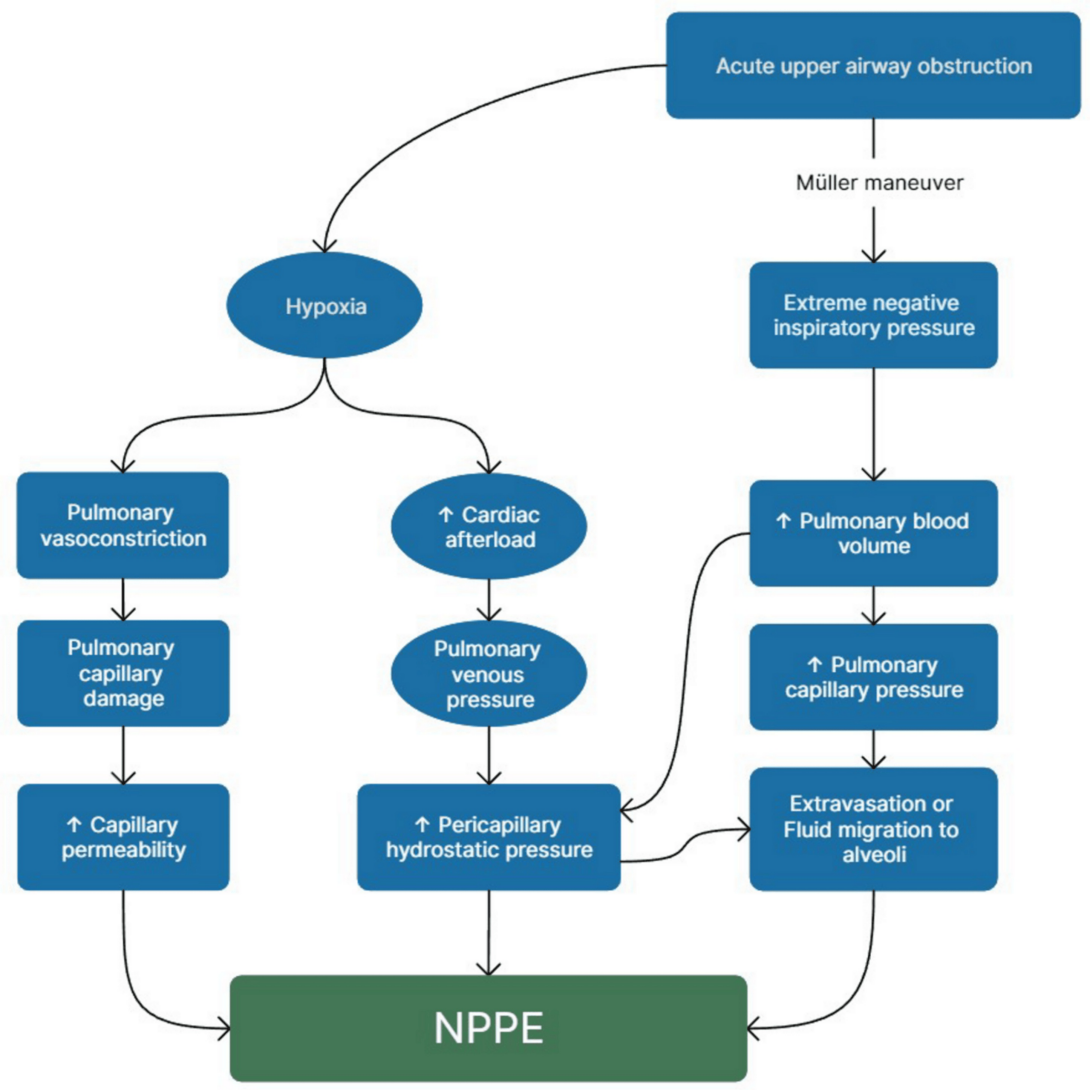
Pathophysiology of negative-pressure pulmonary edema (NPPE). Modified from Bhaskar et al. [17]. Wolters Kluwer Medknow Publications, under the CC BY-NC-SA license.

The main pathophysiology in the occurrence of NPPE is Müller’s maneuver, which is the generation of massive negative intrathoracic pressure by the respiratory muscles and diaphragm against the obstructed or closed glottis [8]. The diaphragm and respiratory muscles recover from the paralyzing effect after the reversal of muscle relaxants, but the laryngeal muscle remains partially under the effect of muscle relaxants, and extubation causes trickling of supraglottic and laryngeal secretions into the larynx, leading to laryngeal spasm and closure of the glottis. This results in the generation of a Müller’s maneuver (Figure 1) [15,16]. The physiopathology of NPPE is described mainly by four mechanisms: (1) increased hydrostatic pressure in pulmonary capillaries, (2) decreased plasma oncotic pressure, (3) increased membrane permeability, and (4) decreased lymphatic drainage from the lung. More detailed physiopathology of NPPE is described in Figure 1.

The normal intrathoracic pressure is -3 to -10 cm H₂O. When the negative thoracic pressure increases abnormally to -50 to -100 cm H₂O due to Müller’s maneuver, a sudden increase in venous return to the heart occurs, namely, to the left ventricle due to a sudden increased load, thus increasing both end-diastolic and end-systolic left ventricular pressure. The sudden increase in pulmonary vascular pressure from low pulmonary pressures, in combination with high left ventricular end-diastolic pressure and lower left ventricular compliance, will cause pulmonary edema [8,17].

Despite intubation and the obstruction being relieved, pulmonary edema takes time to resolve. This is likely because the dramatic increase in venous return may simultaneously reduce cardiac output and impair venous drainage into the left atrium, thus raising pulmonary capillary pressures. At the same time, the interalveolar pressure drop leads to alveolar cell junction disruption, causing a continuous increase of interstitial fluid into the alveolar space, and pulmonary edema persists even if the airway obstruction is resolved [17]. To summarize the pathophysiology of NPPE, the initial inspiratory effort against an obstructed airway generates a deep fall of the pleural pressure from an average value of -4 cm H₂O down to -140 mmHg [8]. The patient forcefully inhales against a blocked airway, generating high negative pressure in the lungs, leading to increased transvascular fluid filtration. This negative pressure increases the pressure difference between the pulmonary capillaries and the surrounding tissues, causing fluid to leak into the interstitial space and alveoli, and leading to fluid accumulation in the lungs and edema formation [13].

The chronic obstruction of the upper airway generates chronic PEEP with an increased end-expiratory lung volume after surgery or therapeutic interventions. The relief of the chronic obstruction causes loss of chronic PEEP, and the increased lung volumes and pressures become normal again, creating a negative intrapulmonary pressure. If this negative pressure rises to a significant level, it leads to fluid accumulation in the interstitium and alveoli, thus causing pulmonary edema [17]. Severe bronchospasm has been reported to cause NPPE [18]. Recently, Kotani et al. reported two cases of tracheostomy tube obstruction causing unexpected NPPE [19].

Diagnosis

The diagnosis of NPPE is established by a clinical event, and radiological evidence can exclude other common etiologies of pulmonary edema. NPPE occurs within a few minutes of extubation, although it has also been reported to occur after a few hours of extubation [20]. Patients typically have signs of upper airway obstruction such as gurgling sounds, stridor, use of accessory muscles of respiration, respiratory distress (tachypnea, dyspnea), oxygen desaturation, hypoxia, cyanosis, and pink frothy secretions upon cough or through the endotracheal tube. Pulse oximetry shows oxygen desaturation, and arterial blood gas analysis reveals hypoxia, hypercapnia, and respiratory acidosis. The severity of the clinical manifestations varies widely in NPPE.

**Figure 2:**
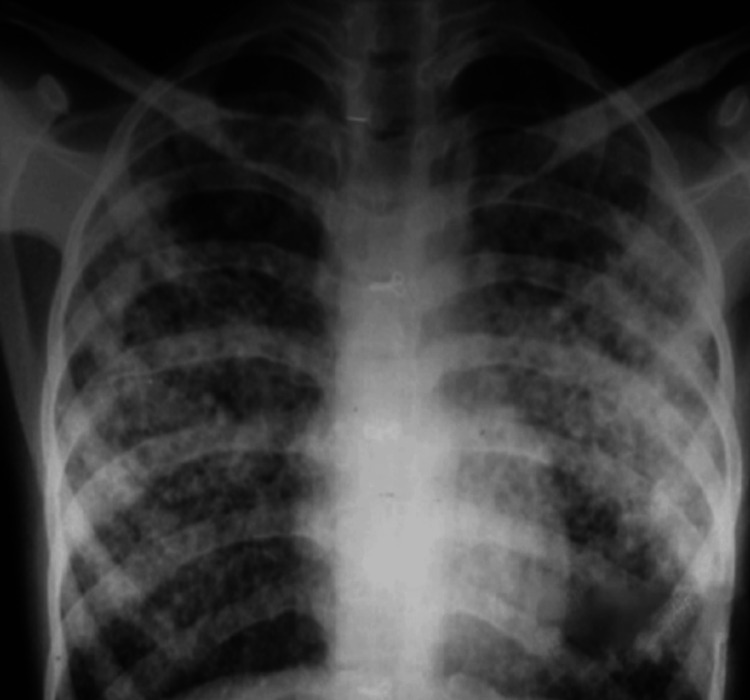
Chest X-ray showing bilateral opacities. Figure obtained from Dr. Nissar Shaikh’s image collection.

Chest X-ray (Figure 2) typically shows diffuse interstitial and alveolar infiltrates, often with a central and non-dependent distribution (more prominent in the middle and upper lung fields). Point-of-care ultrasound is very useful and accurate, and can be done at the bedside; hence, it is increasingly being used to diagnose pulmonary edema, including NPPE [21]. More severe cases may need a chest CT scan, which may show a striking central and non-dependent distribution of ground-glass opacities (edema/hemorrhage), and can help differentiate NPPE from other forms of pulmonary edema [22]. Initially, there can be hemodynamic collapse requiring vasopressor or inotropic support. The patient is usually tachycardic. ECG typically shows signs of myocardial rhythm disturbance or ischemic changes. The echocardiogram is usually normal. Typically, after intubation and release of the upper airway obstruction, the patient shows significant improvement [23].

Differential diagnosis

NPPE should be differentiated from fluid overload, cardiogenic or neurogenic pulmonary edema, aspiration pneumonia, acute respiratory distress syndrome (ARDS), pulmonary embolism, and tension pneumothorax [24]. Table 2 explains how to differentiate these conditions from NPPE. Cardiac issues can be excluded by ruling out acute ischemic heart disease, heart failure, and fluid overload through ECG, echocardiogram, and cardiac enzyme measurements. Brain injury and anaphylaxis are excluded based on clinical assessment and imaging studies and allergic workup, respectively [25].

**Table 2:** Differential diagnosis of negative-pressure pulmonary edema (NPPE).

Variable	Etiology	Diagnosis
Cardiogenic pulmonary edema	Impairment of cardiac function or cardiac dysfunction	Abnormal echocardiography
Aspiration pneumonia	Nausea, vomiting, or both during the perioperative period	History, physical examination, and imaging studies
Pulmonary embolism	Hypercoagulability — risk factors	CT angiography is diagnostic
Volume overload	Large-volume transfusion or surgical procedure	Advanced hemodynamic monitors, showing abnormal higher preload parameters and superior vena cava or inferior vena cava collapsibility index
Neurogenic pulmonary edema	Neurological conditions and emergencies causing increased intracranial pressure	Brain CT scan, MRI, and echocardiography
Tension pneumothorax	Barometric or surgical trauma in the perioperative period	Chest X-ray, ultrasonography of the chest, and clinical examination
Acute respiratory distress syndrome	Risk factors, such as sepsis, pneumonia, and embolism	History, clinical examination, X-ray, and CT scan

Treatment

NPPE is a potentially fatal clinical condition and a perioperative emergency. Patients with NPPE require close monitoring in the intensive care unit (ICU) or post-anesthesia care unit to assess their response to treatment and detect any potential complications. Invasive hemodynamic monitoring is usually not required but can be helpful in the differential diagnosis [26]. The management of NPPE varies according to severity. The primary target in NPPE is to relieve the upper airway obstruction, either by the use of non-invasive ventilation (NIV) or by securing the airway and relieving the obstruction with endotracheal intubation. NIV is an important, commonly used tool to prevent and treat NPPE or acute respiratory failure while avoiding endotracheal intubation. NIV in NPPE reduces the work of breathing and improves gas exchange and alveolar recruitment, thus improving hemodynamics by reducing cardiac overload and increasing cardiac output [23].

The main concerns in NPPE are monitoring, relieving airway obstruction, and oxygenation with supplemental and assisted ventilation. More than 50% of NPPE patients improve with NIV, whereas fewer than 50% require endotracheal intubation [27].

In severe NPPE cases requiring endotracheal intubation and invasive ventilation with PEEP, the endotracheal tube relieves the upper airway obstruction, while invasive ventilation with PEEP opens the alveoli and keeps them open, thus improving oxygenation [27].

The use of diuretics, particularly furosemide, is controversial, as NPPE is not a result of fluid overload. Most of the time, physicians administer furosemide early, suspecting fluid-overload pulmonary edema, but the use of diuretics remains controversial in NPPE cases [28].

As NPPE patients are dehydrated due to preoperative nil by mouth and transfer of large amounts of fluid to the lungs, these may aggravate and deteriorate the patient’s condition, which can progress to acute kidney injury (AKI). Steroids are not recommended in NPPE patients as they will not help address the underlying mechanism of NPPE. Additionally, steroids may cause metabolic issues [13].

The use of bronchodilators with β-agonists appears to be beneficial, as these agents help in clearing the fluid from alveoli, thus aiding patient recovery. Although there is no bronchospasm in NPPE, increased fluid in the alveoli compresses the smaller alveoli. There is not much information available about the duration and dosage of β-agonists to be administered, which depends upon the patient’s severity and recovery [13,23,27].

NPPE may progress to ARDS, not responding to treatment, and may require urgent extracorporeal membrane oxygenation [29]. On the other hand, NPPE may need prolonged weaning and rehabilitation. In these patients, the use of dexmedetomidine may be helpful [30]. Table 3 provides an ICU-focused management algorithm summary of NPPE.

**Table 3:** Summary of the risk factors, diagnosis, pathophysiology, and management of negative-pressure pulmonary edema (NPPE).

Factors	
Risk factors	Post-extubation laryngospasm, young male patients, endotracheal tube obstruction, after removal of upper airway tumors
Diagnosis	NPPE is diagnosed by clinical manifestations, point-of-care ultrasound, chest X-ray, and severe cases with CT scans
Differential diagnosis	NPPE can be differentiated by history, clinical manifestations, risk factors, advanced hemodynamic monitoring, and point-of-care ultrasound
Pathophysiology	Generation of extreme negative pressure is the foremost etiology in type 1 NPPE, and removal of chronic upper airway obstruction causes type 2 NPPE
Treatment	Most NPPE cases respond to non-invasive ventilation therapy and a lesser number of NPPE patients need invasive ventilation and supportive intensive care therapy
Prognosis	NPPE has a lower morbidity and mortality if diagnosed promptly and managed immediately

Morbidity and mortality

Most NPPE cases rapidly improve with care within 24 to 48 hours. In cases of nasopharyngeal surgeries, the majority of NPPE patients recover in 33 hours, and 6% of NPPE patients develop long-term morbidity, mainly ARDS and AKI due to diuretic overuse or abuse. Patients may have a more complicated course with difficult weaning and even recurrent pulmonary edema. The use of dexmedetomidine for weaning from mechanical ventilation has been reported to be successful in patients with NPPE [25]. Dexmedetomidine is a highly selective α2-adrenoceptor agonist that has been increasingly used in critically ill patients [31]. NPPE has a mortality rate of 5%. Reports on mortality also vary. Rarely, patients with NPPE may develop long-term complications, including myocardial infarction, transient ischemic attack, non-ST-elevation myocardial infarction, hypoxic brain injury, pulmonary hemorrhage, septic shock, and cardiac arrest [24]. The mortality rate of NPPE has previously been described as 11% to 40%, with a more recent literature review reporting a mortality rate of only 2% [13,32]. Extremes of age and increased hospital stay are significant risk factors for morbidity in NPPE patients [25].

Prevention

Complete prevention of NPPE may not be possible. A few strategies suggested in the literature to prevent NPPE are aspiration of oropharyngeal secretions before extubation or administration of lidocaine. Dexamethasone before extubation may reduce laryngeal edema, irritation, and spasm [33]. For extubation after completion of surgery, clinical assessment alone is insufficient. Neuromuscular blockade should be monitored by the train-of-four ratios, which help determine the complete reversal of muscle paralysis. A ratio of 0.9 may indicate muscle recovery and may prevent the occurrence of NPPE [34]. Ensuring adequate anesthesia during procedures, especially those involving the airway, can help prevent laryngospasm and subsequent NPPE [35,36]. Extubating patients when they are either deeply anesthetized or fully awake with a clear airway can reduce the risk of laryngospasm [13]. In high-risk patients with repeated NPPE, the use of prophylactic continuous positive airway pressure may be considered [12]. Recurrent NPPE should be kept in mind while administering general anesthesia in a young patient, and the patient should be specifically asked whether a history of any such occurrence exists. Any positive history should prompt the physician to take prophylactic measures to prevent recurrent NPPE and decrease the associated morbidity. Early recognition allows prompt application of positive airway pressure and rapid resolution [13].

## Conclusions

NPPE, the occurrence of pulmonary edema after extubation of the trachea, is a diagnosis of exclusion. It is a life-threatening clinical entity. Müller’s maneuver, or excessive negative pressure against a partially or fully obstructed glottis, results in increased negative intrathoracic pressure. The risk factors include post-extubation laryngospasm, young male patients, endotracheal tube obstruction, and removal of upper airway tumors. NPPE is diagnosed by the presence of pink frothy sputum and confirmed by imaging studies. NPPE is treated by relieving the airway obstruction, NIV, or invasive ventilation with PEEP. Many of these patients receive diuretics, but as this can aggravate perioperative dehydration, it is not recommended. The use of β-agonist medications is effective, as they help clear the alveoli quickly. Steroids are not recommended in the treatment of NPPE. NPPE improves within 24 to 48 hours; however, a few patients may progress to ARDS, requiring a prone position and longer ICU stay. NPPE may be prevented by clearing secretions before extubation and extubating patients after full neuromuscular recovery, monitored by a train-of-four ratio of 0.9 or 1. Ensuring adequate anesthesia during procedures and extubating patients when they are either deeply anesthetized or fully awake reduces the risk of laryngospasm. In high-risk patients with repeated NPPE, early recognition and application of prophylactic non-invasive ventilation are beneficial.
